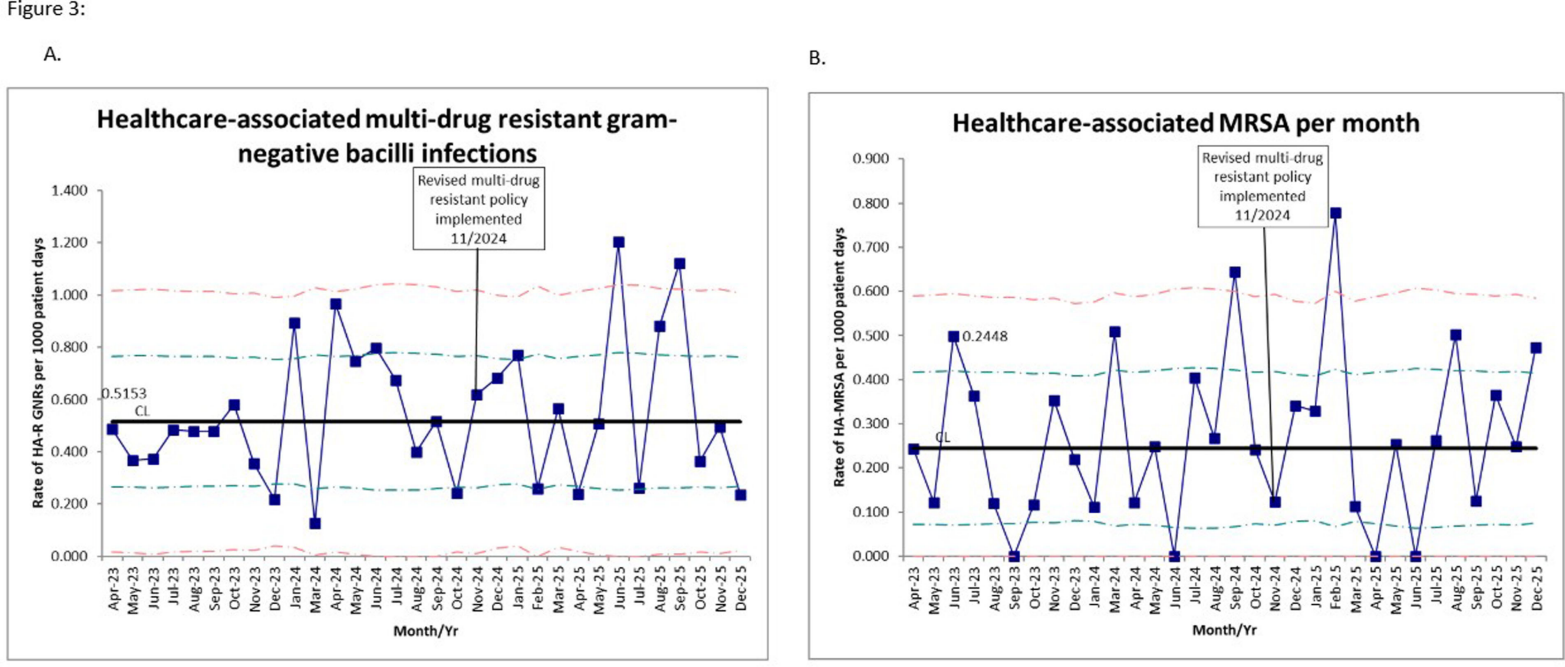# 350 Implementation of Antimicrobial Stewardship Interventions in Community Health Clinics

**DOI:** 10.1017/ash.2026.10689

**Published:** 2026-06-23

**Authors:** Matthew McHugh, Nabgha Farhat, Anna ODonnell, Caitlin Li, Larry Kociolek, Ayelet Rosenthal

**Affiliations:** 1 Ann & Robert H. Lurie Children’s Hospital of Chicago; 2 Lurie Children’s Hospital; 3 Ann & Robert H. Lurie Childrens Hospital of Chicago; 4 Lurie Childen’s Chicago; 5 Lurie Children’s Hospital of Chicago, Northwestern University

## Abstract

**Background:** Hospitalized patients colonized or infected with multidrug-resistant organisms (MRO) have historically been placed on contact precautions (CP) for months or indefinitely. However, the necessity of CP for MROs to prevent nosocomial transmission is controversial, and adult data show that standard precautions might suffice for select MROs. Data in pediatrics are lacking, and isolation practices vary between institutions. After literature review and benchmarking with peer institutions, we sought to decrease the amount of time patients in our institution with MROs spent on CP. We aimed to decrease the proportion of inpatient days on CP for patients with MROs while maintaining or decreasing the rate of healthcare associated (HA)-MROs. **Methods:** Between -12/, we completed a series of plan-do-study-act cycles. These included (1) major MRO policy update with new de-isolation criteria (Table 1), (2) frontline provider education, and (3) implementation of a secondary clinical review by infection prevention and control (IP&C) medical director, to appropriately expedite the removal of undue isolation of patients with MRO infection status. Eventually, infection preventionists no longer needed a secondary review, so the fourth PDSA cycle eliminated that secondary review. Our outcome measure was proportion of inpatient days in CP for patients with MROs. Our process measure was the number of medical director case reviews/month; balancing measure was number of total HA-MRO, HA resistant gram-negative rods (GNR) and HA Methicillin-resistant Staphylococcus aureus (MRSA)/month. Data were monitored on statistical process control charts. **Results:** Proportion of MRO isolation days decreased from 4.5% to 2.6% (figure 1). Number of medical director secondary reviews increased and was sustained for 8-months until no longer required (figure 2). No center line shifts were noted in the?rate of HA-MROs (figure 3). **Conclusion:** Shortening the duration of CP for select MROs can be safely implemented and did not increase HA-MDRO rate in our children’s hospital.

**Figure:**
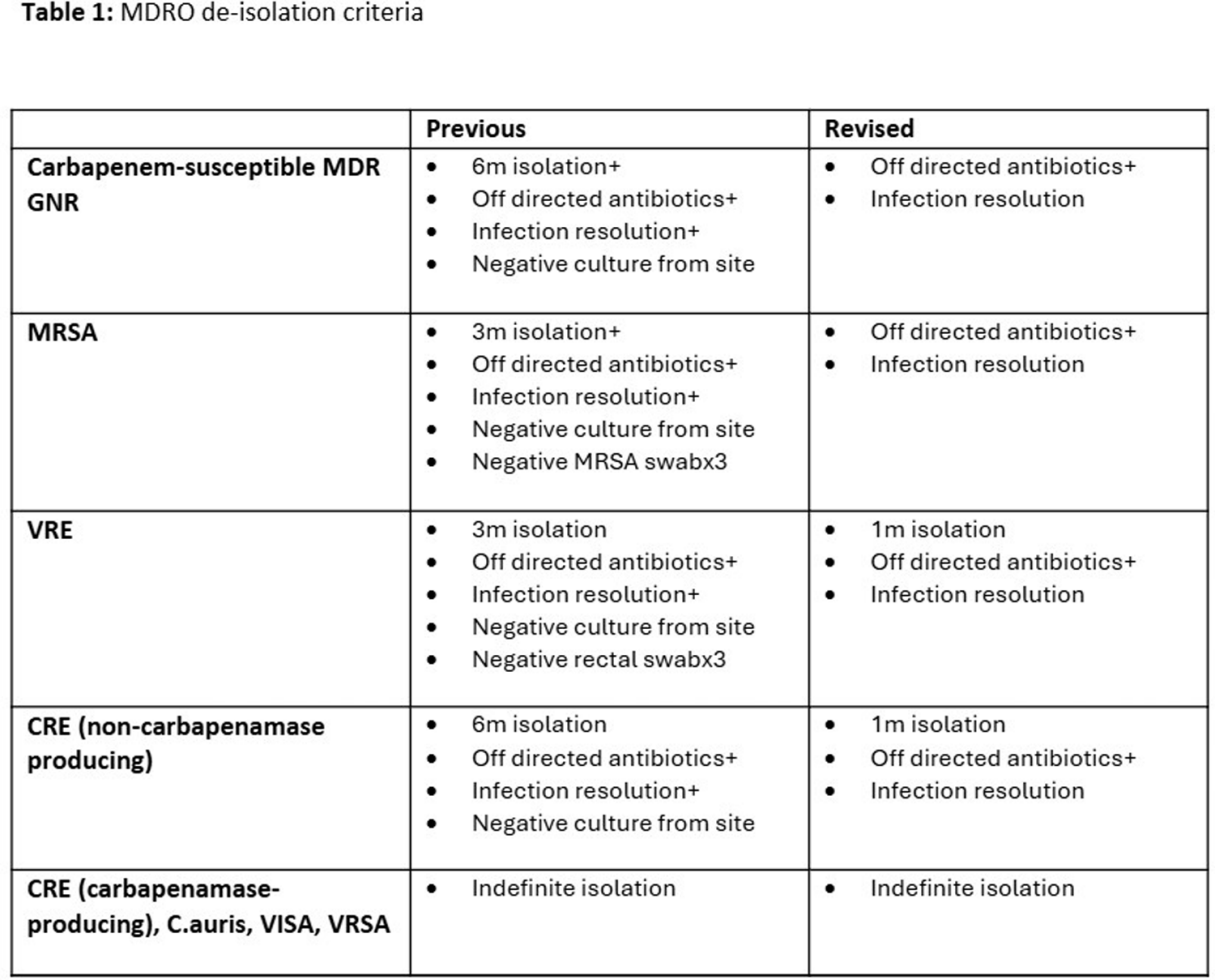

**Figure:**
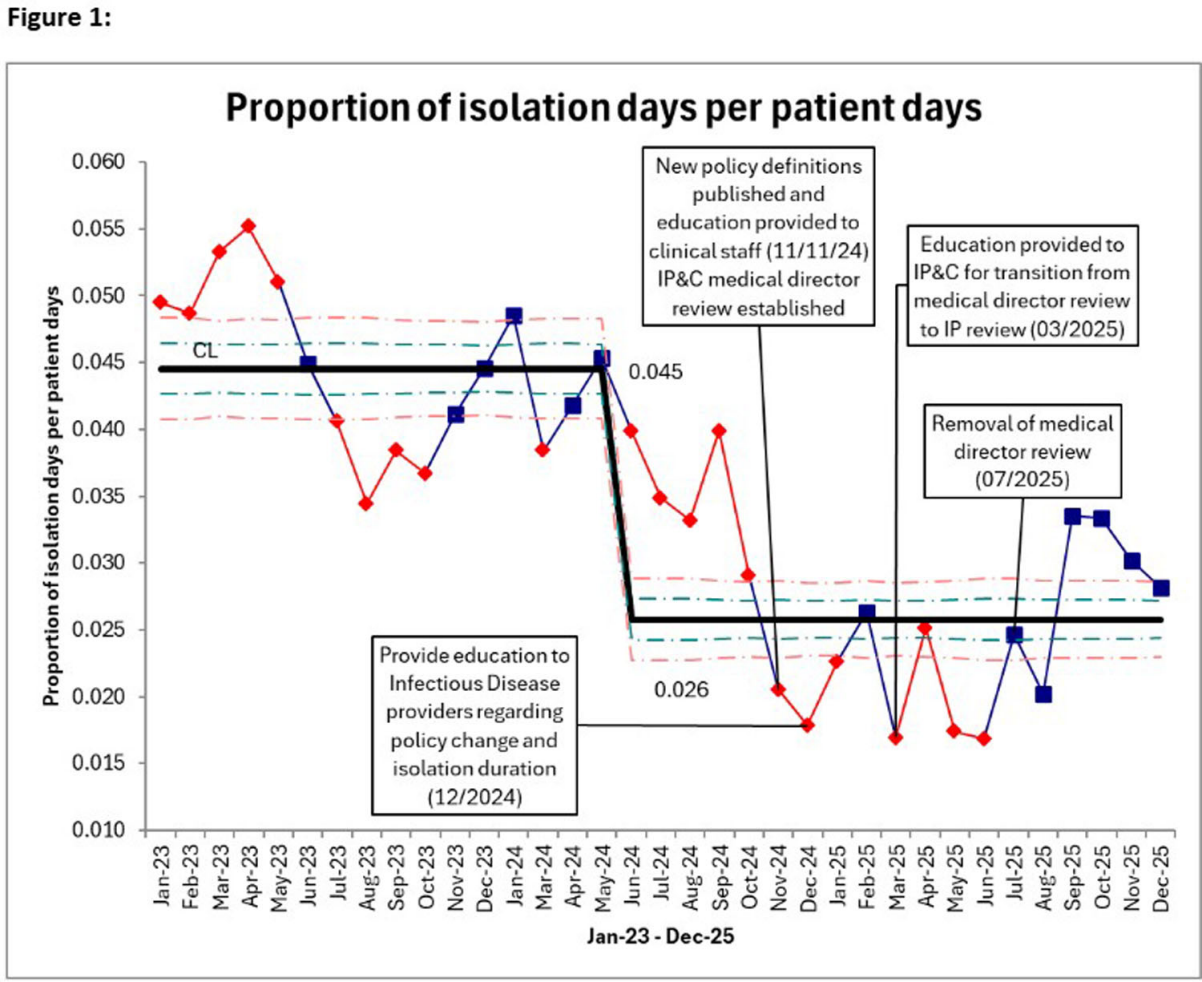

**Figure:**
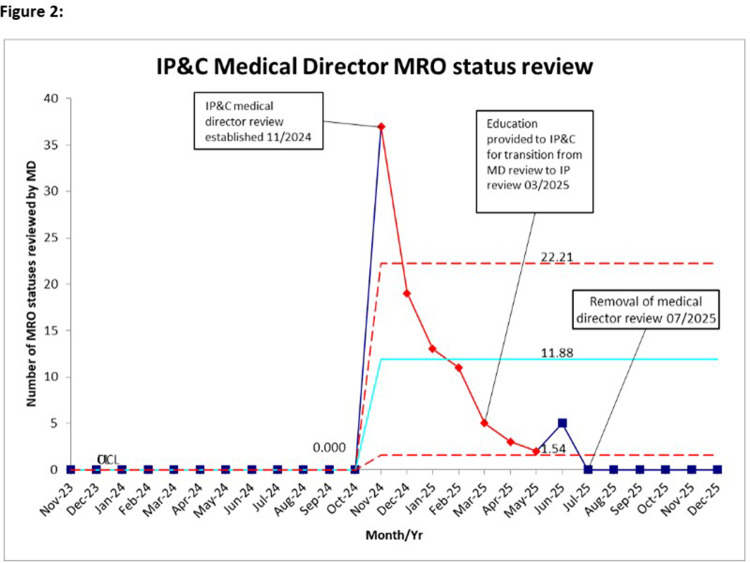

**Figure:**